# Introduction of ROSA robotic-arm system for total knee arthroplasty is associated with a minimal learning curve for operative time

**DOI:** 10.1186/s40634-022-00524-5

**Published:** 2022-08-30

**Authors:** Scott M. Bolam, Mei Lin Tay, Faseeh Zaidi, Raghavendra P. Sidaginamale, Michael Hanlon, Jacob T. Munro, A. Paul Monk

**Affiliations:** 1 Department of Orthopaedic Surgery, Auckland City Hospital, Auckland, New Zealand; 2 Department of Surgery, Faculty of Medical and Health Sciences, University of Auckland, Building 502 20185 Park Road, Grafton, 1023 Auckland, New Zealand; 3 Department of Orthopaedic Surgery, North Shore Hospital, Auckland, New Zealand; 4 Auckland Bioengineering Institute, University of Auckland, Auckland, New Zealand; 5 Department of Orthopaedics, University Hospital of North Tees, Stockton on Tees, United Kingdom

**Keywords:** Learning curve, Operative time, Robotically assisted system, Personalised robotic TKA, ROSA knee system

## Abstract

**Purpose:**

The introduction of robotics for total knee arthroplasty (TKA) into the operating theatre is often associated with a learning curve and is potentially associated with additional complications. The purpose of this study was to determine the learning curve of robotic-assisted (RA) TKA within a multi-surgeon team.

**Methods:**

This prospective cohort study included 83 consecutive conventional jig-based TKAs compared with 53 RA TKAs using the Robotic Surgical Assistant (ROSA) system (Zimmer Biomet, Warsaw, Indiana, USA) for knee osteoarthritis performed by three high-volume (> 100 TKA per year) orthopaedic surgeons. Baseline characteristics including age, BMI, sex and pre-operative Kellgren-Lawrence graded and Hip-Knee-Ankle Axis were well-matched between the conventional and RA TKA groups. Cumulative summation (CUSUM) analysis was used to assess learning curves for operative times for each surgeon. Peri-operative and delayed complications (infection, periprosthetic fracture, thromboembolism, and compromised wound healing) and revisions were reviewed.

**Results:**

The CUSUM analysis for operative time demonstrated an inflexion point after 5, 6 and 15 cases for each of the three surgeons, or 8.7 cases on average. There were no significant differences (*p* = 0.53) in operative times between the RA TKA learning (before inflexion point) and proficiency (after inflexion point) phases. Similarly, the operative times of the RA TKA group did not differ significantly (*p* = 0.92) from the conventional TKA group. There was no discernible learning curve for the accuracy of component planning using the RA TKA system. The average length of post-operative follow-up was 21.3 ± 9.0 months. There was one revision for instability in the conventional TKA group and none in the RA TKA group. There were no significant difference (*p* > 0.99) in post-operative complication rates between the conventional TKA and RA TKA groups.

**Conclusions:**

The introduction of the RA TKA system was associated with a learning curve for operative time of 8.7 cases. Operative times between the RA TKA and conventional TKA group were similar. The short learning curve implies this RA TKA system can be adopted relatively quickly into a surgical team with minimal risks to patients.

**Supplementary Information:**

The online version contains supplementary material available at 10.1186/s40634-022-00524-5.

## Introduction

Total knee arthroplasty (TKA) is frequently performed to control pain, restore function and enhance the quality of life for patients with end-stage osteoarthritis [7]. But despite this, between 10 and 20% of patients are not satisfied after surgery and continue to have post-operative pain and functional limitations, with associated implications for recovery [5, 6]. Surgical errors in implant positioning and soft-tissue balance can result in early failure following TKA. Recent advancements in surgical technology have led to the introduction of robotic-assisted (RA) surgery, with several different systems available for surgeons to minimise errors due to implant positioning [28].

The ROSA® (Robotic Surgical Assistant) Knee system (Zimmer Biomet, Warsaw, Indiana, USA) for TKA was recently introduced. ROSA can be considered collaborative robotics, where the surgeon remains in charge of the procedure but collaborates with a smart robotic tool to perform the surgery with high accuracy and reproducibility [2, 21, 22]. The advantages of ROSA compared with other RA TKA include: requiring only radiographs for pre-operative planning, a collaborative robotic system where the robot completes the surgeon skills, and predictive robotics with machine learning incorporated into the system [2, 13].

A learning curve of operative times has been found with the introduction of RA TKA surgery that was variable based on surgeon experience and volume [9, 18, 30]. Reporting of learning curves associated with introducing a novel system will enable surgeons to better understand the impact of implementing RA TKA on their surgical workflow, facilitate operative planning and scheduling, and understand risks of complications before surgical proficiency [10, 26].

Most previous studies have examined learning curves in other RA TKA systems and reported highly variable learning curves (6 to 43 cases) between different centres [4, 9, 18, 20, 23, 26, 31]. To date, only one previous study [30] has examined the learning curve of the ROSA TKA system that was performed at a single-centre with a minimal postoperative follow-up of 3 months. Previously, the introduction of new RA TKA systems has been associated with increased operative time and higher rates of adverse events for cases performed during the initial learning phase, including pin site periprosthetic fracture in the femur and tibia and pin site infections [9, 18, 25, 30, 31]. A recent systematic review reported the overall incidence of pin-related fractures with computer navigated and RA TKA to range from 0.06% to 4.8% [25]. Therefore, it is important for further studies at different international centres to evaluate the learning curve associated with the introduction of the ROSA robotic system.

We hypothesised that: [1] cumulative experience with ROSA TKA would lead to improved operative times; [2] surgeon’s learning curve would be more variable and longer than previously been reported in the current literature; and [3] the introduction of the ROSA TKA system would be associated with higher rates of robot-related complications including pin site infection and fractures.

The main aim of this study was to determine the learning curve of the ROSA system for TKA for a multi-surgeon team. The secondary aims were to compare the operative time of patients undergoing ROSA TKA versus conventional TKA and to identify any complications associated with the introduction of this RA system.

## Materials and methods

Ethics approval was obtained from the institutional ethical committee for this study (#000,072). The ROSA platform (Zimmer Biomet, Warsaw, Indiana, USA) was introduced into the department in February 2020. This was a single-centre, multi-surgeon study in a public hospital setting with prospectively collected data between February 2020 and November 2021. All surgeons who performed at least 10 RA TKA procedures during the study period were included. The patient cohort was consecutive patients undergoing primary TKA for osteoarthritis. Exclusion criteria were: conversion from unicompartmental knee arthroplasty to TKA, infection, neurological dysfunction limiting knee mobility, or post-traumatic osteoarthritis with severe knee deformity.

Using the same inclusion and exclusion criteria, patients who underwent conventional TKA surgery by the same surgeons in the centre between April 2019 and November 2021 were included as a control group. All surgeons contributed at least 20 conventional TKA cases to the control group. Pre-operative osteoarthritis was grading using the Kellgren Lawrence (KL) classification [14]. Patient pre-operative characteristics are summarised in Table 1.

**Table 1:** Characteristics of patients undergoing RA TKA and conventional TKA groups

	RA TKA	Conventional TKA	Significance
Total	53	83	
Patients	52	80	
Knees	53	83	
Gender	19 (36%)	30 (36%)	NS (> 0.99)
Age (year ± SD)	70.3 ± 8.6	70.5 ± 9.1	NS (0.92)
BMI (kg/m^2^ ± SD)	31.8 ± 5.8	31.3 ± 6.3	NS (0.58)
Left side	32 (60%)	39 (47%)	NS (0.18)
Pre-operative KL grade			NS (0.23)
Grade 3	10	24	
Grade 4	43	59	
Pre-operative HKA (° ± SD)	-7.0° ± 7.2	-4.1° ± 9.9	NS (0.11)

*BMI* Body mass index, *HKA* Hip-Knee-Ankle Axis, (Varus < 0°; Valgus < 0°), *KL* Kellen-Lawrence, *NS* Not significant, *SD* Standard deviation, *TKA* Total knee arthroplasty

### Operation details

The operations were performed by three surgeons who were fellowship-trained high-volume (> 100 TKA per year) arthroplasty surgeons [16]. All surgeons received 4 h of theoretical training with the ROSA system for TKA but had not performed any RA TKA surgery with the system prior to study. A subvastus approach was used in all cases. The Persona (Zimmer Biomet, Indiana, USA) cruciate-retaining (CR) or posterior stabilised (PS) implant was utilised. A single midline skin incision was utilised for all TKA that incorporated the femoral and tibial pins. Two femoral pins (3.2 mm diameter) were positioned in the proximal wound underneath the vastus medialis. Two tibial pins (3.2 mm diameter) were positioned in the distal wound in the anteromedial tibial crest.

### Outcomes measures

A retrospective review of patient clinical notes, radiographs and intra-operative data was performed by three authors (SMB, MLT and FZ).

### Operative time

For this study, the operative time was defined as the time between the initial skin incision to final wound closure. The operative time was extracted from the electronic operation note file, which was completed at the time of surgery.

### Complications

All patient clinical notes were reviewed for complications related to the ROSA robot (including fractures of the femur or tibia due to pin placement, superficial/deep infections at the pin tracts) and other non-robotic complications related to TKA. This included peri-operative and delayed complications (wound infection, periprosthetic fracture, thromboembolism, and compromised wound healing) and revisions that was last checked on July 10 2022.

### Statistical analysis

The learning curves for the operative time were analysed using a Cumulative Summation (CUSUM) method, as previously described [9, 10, 29]. CUSUM analyses were set using the overall mean values of the operative times in the RA TKA group. CUSUM values were the cumulative difference between each data point and an overall mean value. An inflexion point in the visualised trend was defined as the transition from the learning to proficiency phases.

Operative time was used as the primary outcome measure for sample size calculation using previously published data on operative times with similar surgical techniques for TKA. The minimal clinical difference was set at 6 min and standard deviation (SD) at 10 min [24]. This study required 44 patients in each arm to detect a minimum difference in operative time using a two-tailed, two-sample t-test with a power of 80% and significance level of 5% [8].

Statistical differences were determined with Fisher’s exact test (categorical data), t-tests or one-way ANOVA (normally distributed continuous variables) with post-hoc Tukey test or Mann–Whitney and Kruskal–Wallis tests (non-parametric continuous variables). Data are presented as mean ± standard deviation (SD). Statistical analyses were performed with PRISM 8 (GraphPad, San Diego, CA). A p-value < 0.05 was considered significant**.**

## Results

A total of 132 patients undergoing 136 TKA were included in the present study. Of these, 53 were RA TKA, and 83 were conventional TKA. The RA TKA group did not show statistically significant differences (*p* > 0.05) in age, BMI, gender, pre-operative KL grade and pre-operative Hip-Knee-Ankle Axis (HKA)compared to the conventional TKA group (Table 1).

### Operative times

**Fig. 1:**
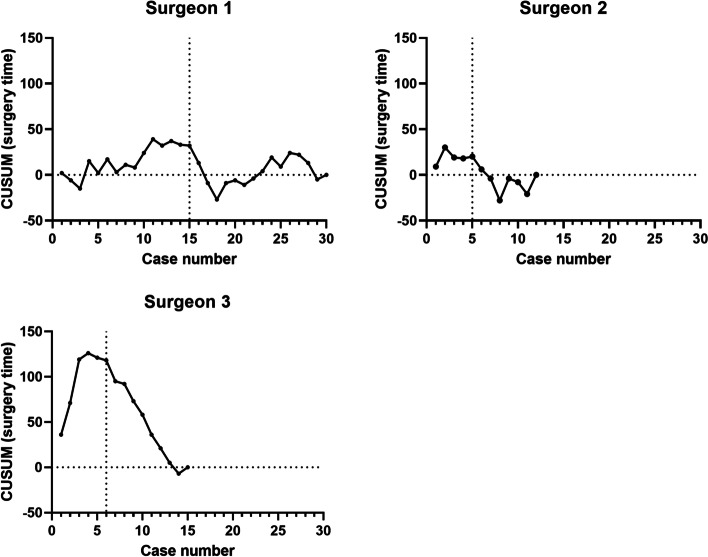
Cumulative Summation (CUSUM) analysis of the initial RA total knee arthroplasty cases of three surgeons. Inflexion points were observed at 15, 5 and 6 cases for Surgeons 1, 2 and 3, respectively

For RA TKA cases, the CUSUM analyses demonstrated a clear inflexion point after 15, 5 and 6 cases for Surgeons 1, 2 and 3, respectively. The inflexion point was used to identify two phases in the learning curve the learning phase (before inflexion point) and the proficiency phase (after inflexion point) (Fig. 1).

The learning phase of RA TKA was associated with 4 min longer operative time (114 ± 17 min); however, this was not significantly different compared with the proficiency phase (110 ± 20 min, *p* = 0.53; Table 2). There were no significant differences (*p* > 0.05) in age, BMI, gender, pre-operative haemoglobin (Hb) level, post-operative Hb change ore pre-operative HKA for patients in the learning and proficiency groups (Table 2).

**Table 2:** Comparison of patient demographics within the learning curve phases of ROSA TKA

Patient characteristic	Learning phase (*n* = 26)	Proficiency phase (*n* = 27)	*p*-value (Learning vs proficiency)
Age (years)	70.0 ± 10.5	70.6 ± 6.4	NS (0.80)
BMI (kg/m^2^)	30.8 ± 5.6	32.8 ± 6.0	NS (0.22)
Male gender	10 (39%)	9 (33%)	NS (0.92)
Preoperative Hb (g/L)	134.6 ± 11.7	139.6 ± 13.1	NS (0.15)
Postoperative Hb change (g/L)	22.3 ± 8.1	26.9 ± 10.1	NS (0.07)
Pre-operative HKA (° ± SD)	-4.8 ± 7.8	-8.6 ± 6.4	NS (0.06)
Operative (skin to skin) time (mins)	114.0 ± 17.3	110.8 ± 19.6	NS (0.53)

*BMI* Body mass index, *Hb* Haemoglobin, *HKA* Hip-knee-ankle axis (Varus: > 0°; Valgus < 0°), *NS* Not significant, *SD* Standard deviation, *TKA* Total knee arthroplasty

**Fig. 2:**
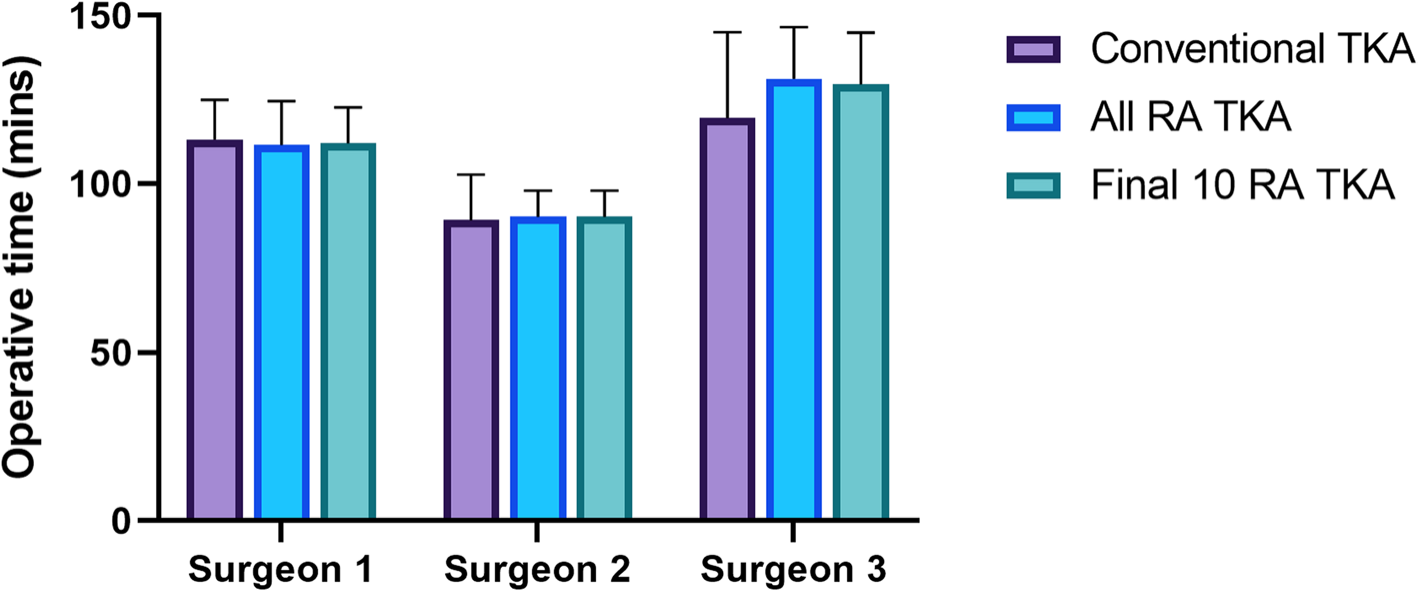
Operative times for conventional TKA versus all RA TKA vs the final 10 RA TKA cases. No significant differences were detected between groups

Furthermore, the operative times of all of the RA TKA groups did not differ significantly from the conventional TKA group (107 ± 16 vs. 111 ± 22 min, respectively, *p* = 0.92). Similarly, the final 10 RA TKA were not significantly different to the conventional TKA group (110 ± 20 min, *p* = 0.98) (Fig. 2).

**Fig. 3:**
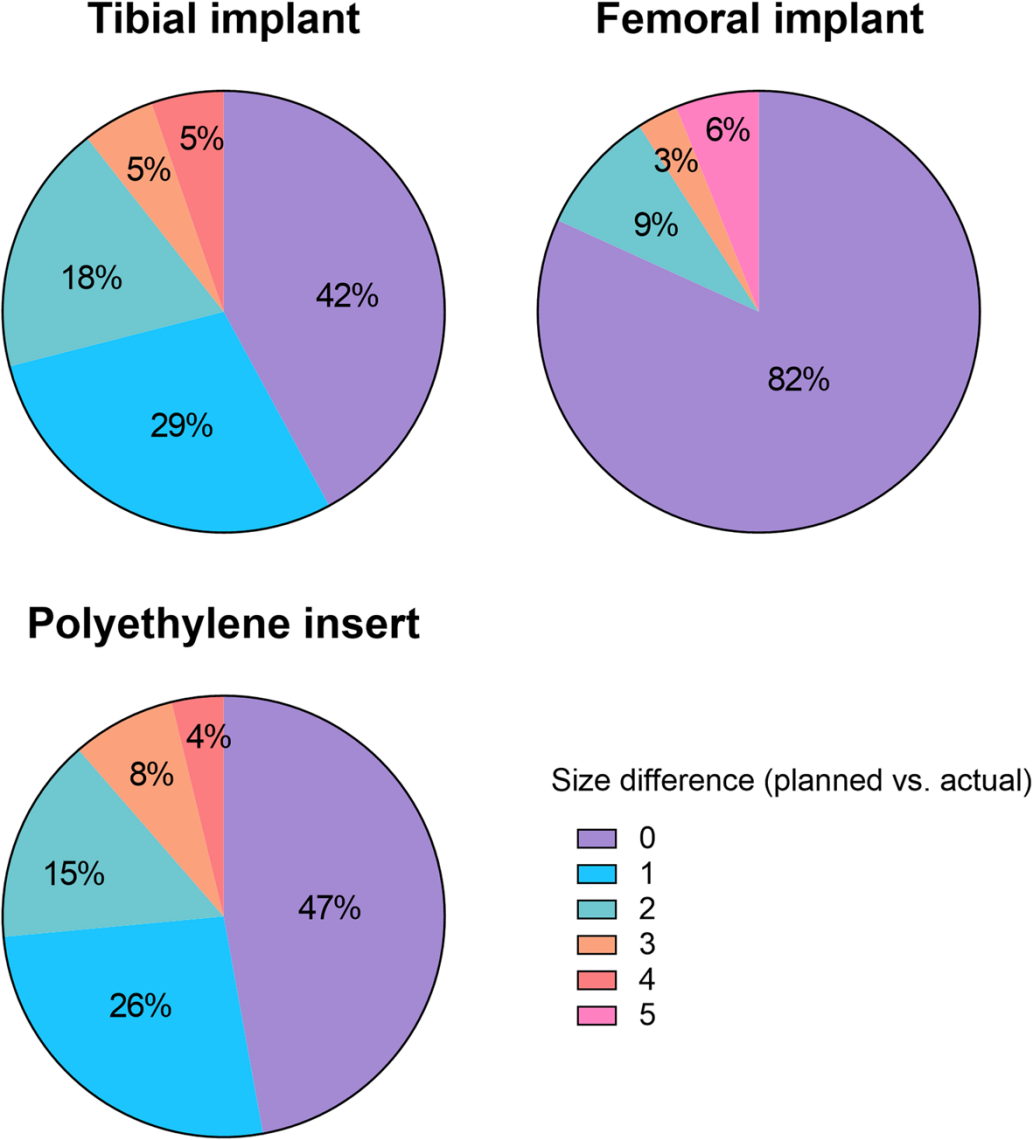
Comparison of implant component planning accuracy with ROSA TKA (planned vs. actual)

Comparison of planned versus actual implant during RA TKA showed that planning was accurate 42% of the time for the tibial implant, 82% of the time for the femoral implant and 47% of the time for the polyethylene insert (Fig. 3). Out of all cases, 10% of tibial implants, 9% of femoral implants and 12% of polyethylene inserts deviated by more than 2 sizes. No discernible learning curve was detected for the accuracy of component planning (Supplementary Data 1).

### Complications

The average length of post-operative follow-up was 21.3 ± 9.0 months (range 3–39 months).There were no complications that were directly related to the RA TKA system, including no pin site fracture or pin site infections.

In terms of other non-robotic complications, in the conventional TKA group, there was one revision indicated for mid-flexion instability at 5 months that was subsequently underwent a polyethylene liner exchange at 12 months. In the RA TKA group, there were no revisions. In the conventional TKA group there were and six post-operative complications (three superficial wound site infections, one post-operative deep vein thrombosis and one post-operative stroke In the RA TK group there were four post-operative complications (two superficial wound site infections, one post-operative pulmonary embolus, and one post-operative death secondary to an unrelated cardiovascular event that occurred 3 months after surgery). There was no significant difference (*p* > 0.99) in the rate of post-operative complications between the conventional TKA and RA TKA groups.

## Discussion

The most pertinent finding of this study was that there was a learning curve of between 5 and 15 cases associated with the introduction of this RA TKA system. Operative times in the learning and proficiency phases were not significantly different. There was no significant increase in operative time in the RA TKA group compared to the conventional TKA group. There were no post-operative complications associated with the introduction of this system. Understanding the learning curve of a novel RA TKA system is an important step in understanding the impact of introducing this procedure into the surgical workflow.

Only one previous study [30] has reported on the learning curve of the ROSA TKA system using the operative time of three surgeons. The authors found a learning curve of between 6 to 11 cases using CUSUM analysis, which was similar to our finding. Other studies have reported on other optically guided RA systems, including the MAKO RIO system (Stryker, Kalamazoo, MI) [9, 20, 31] and reported similar learning curves with operative time, despite some differences in design. In the previous study using the ROSA TKA system, time neutrality between ROSA TKA and conventional TKA was never achieved, although the difference in operative time fell from 29 to 13 min in the learning and mastered phases, respectively. In this study, there was no difference in operative times in ROSA TKA compared to those with a conventional jig-based TKA. In theory, RA TKA surgery should allow a more streamlined surgical approach by decreasing the need for alignment guides and cutting blocks and reducing the need for implant trialling [2, 21, 27]. Other studies with different RA TKA systems have found no difference between RA TKA and jig-based TKA after an initial learning phase [19, 31].

Previous studies have reported learning curves associated with the introduction of different RA TKA ranging from 6 to 43 cases [4, 9, 18, 20, 23, 26, 30, 31]. The learning curve identified in this study was much shorter than previous studies, perhaps because all surgeons were high volume arthroplasty surgeons (> 100 TKA per year) [1, 19]. However, other factors related to the real-life study setting in a public hospital; including different surgical teams for all RA TKA cases of each surgeon [31] and periodic complete suspension of operating due to the Coronavirus Disease 2019 (COVID-19) pandemic [3, 15] did not translate into a longer learning curve. This has important clinical relevance when considering the introduction of this system with other surgeons as it suggests it will have minimal impact on surgical workflow.

Component planning accuracy was achieved between 42 and 82% of the time with this RA TKA system. Actual implant sizes deviated from pre-operative plans by more than two sizes in 10% of cases. No learning curve was discernible. Only one other study to date reported on the accuracy of implant planning with a RA TKA system. In the previous study, there was no learning curve with tibial or femoral component planning with the MAKO system for unicompartmental knee arthroplasty. However, in contrast to the findings reported here, a learning curve was found with polyethylene insert planning for the first 10 cases performed [29]. Achieving correct component sizing in knee arthroplasty is important as this can influence post-operative outcomes and implant survival [32, 33].

The adoption of novel technology may pose a risk of increased adverse events, and it is important to consider outcomes associated with safety. A known complication of RA surgery is the risk of pin-site stress fractures and infections [12, 31]. In this study, the use of the ROSA system did not lead to any complications, adverse events or revision in the patient population, supporting previous findings using other RA TKA systems [9, 31].

There were some limitations with this study that should be noted. Firstly, there were three COVID-19 lockdowns during the study period, which affected the elective surgery caseloads and, subsequently, the learning curve reported [3]. Secondly, while we analysed the learning curve on implant component sizing, we were unable to report on the learning curve associated with component positioning. However, other studies have reported on component positioning and limb alignment with the use of robotic systems [11, 17, 22, 30]. Third, we have reported that there were no adverse events associated with the introduction of the system in this centre. However, it should be noted that this study had a relatively short follow-up of 21 months. Finally, this study was conducted at one site, and the surgeons included in the study were fellowship-trained high-volume surgeons. More research in other centres may be necessary to generate more generalisable findings across different surgical centres and in surgeons with less experience and lower caseloads.

While there was a learning curve associated with introducing the ROSA TKA system, this was relatively short and did not lead to increases in operative time or any additional complications or adverse events for cases performed during the learning phase. The findings from this study suggest that the ROSA system can be implemented with minimal impact on surgical workflows. Understanding the learning curve of a novel RA TKA system is an important step in understanding the impact of adopting this technology into the surgical workflow.

## Supplementary Information

**Additional file 1.** **Supplementary Material 1.** Changes in component sizing (pre- vs. post-plan), per 5 surgeon cases, for [**A**] Tibial implant, [**B**] Femoral implant, and [**C**] Polyethylene insert.

## Abbreviations

BMI: Body mass index
COVID-19: Coronavirus Disease 2019
CUSUM: Cumulative summation
Hb: Haemoglobin
HKA: Hip-Knee-Ankle Axis
KL: Kellgren-Lawrence
RA: Robotic-assisted
ROSA: Robotic Surgical Assistant
SD: Standard deviation
TKA: Total knee arthroplasty
