# Supplementary material for: Introduction of ROSA robotic-arm system for total knee arthroplasty is associated with a minimal learning curve for operative time
Source: J Exp Orthop. 2022 Aug 30;9:86. doi: 10.1186/s40634-022-00524-5 (PMC9427173; doi:10.1186/s40634-022-00524-5)

## Supplementary material

Supplementary Material 1. Changes in component sizing (pre- vs. post-plan), per 5 surgeon cases, for [A] Tibial implant, [B] Femoral implant, and [C] Polyethylene insert.


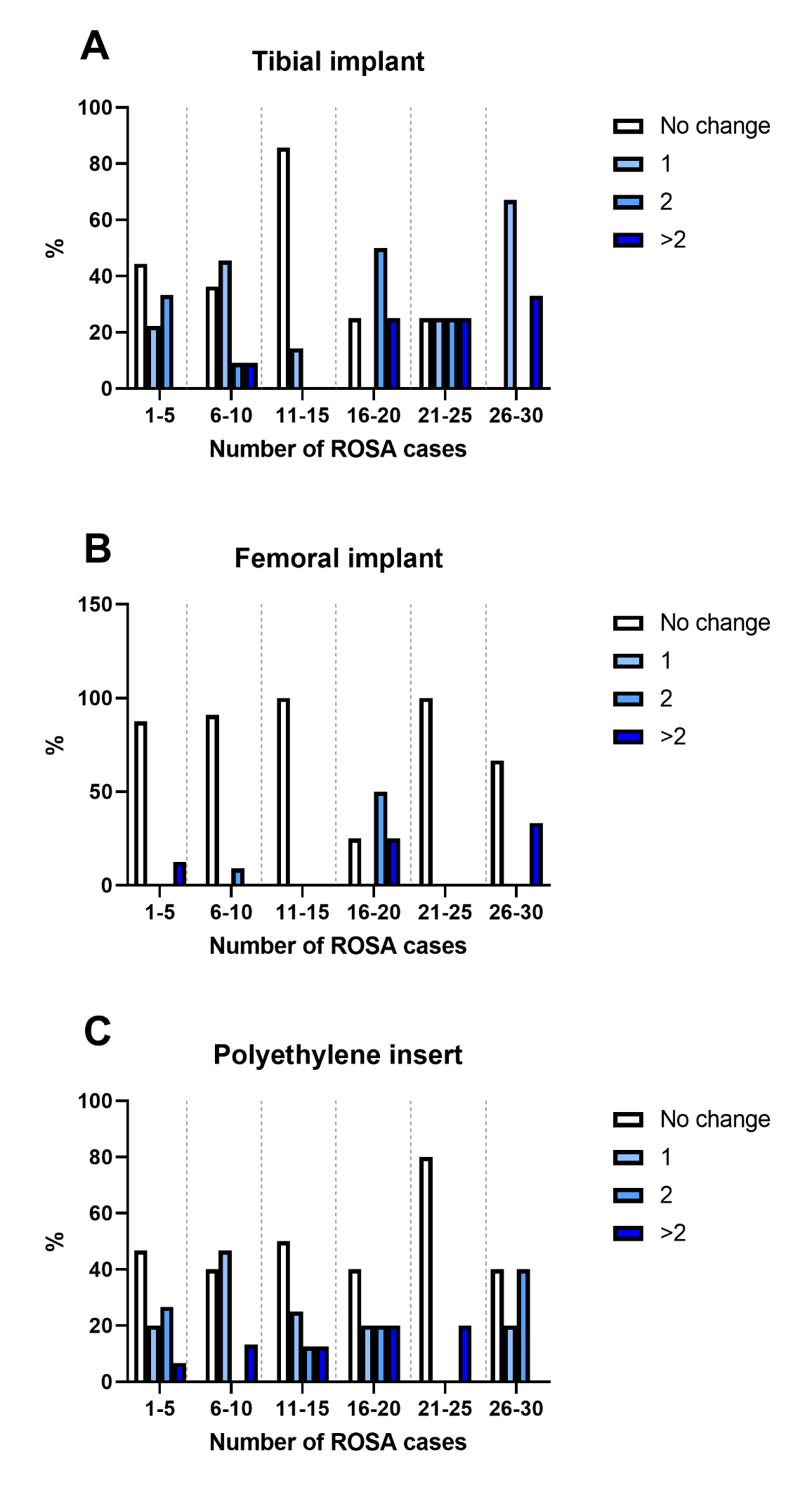

Supplement: Supplementary file 1 — Additional file 1. Supplementary Material 1. Changes in component sizing (pre- vs. post-plan), per 5 surgeon cases, for [A] Tibial implant, [B] Femoral implant, and [C] Polyethylene insert. [file 40634_2022_524_MOESM1_ESM.docx]
